# Comparison of TAPP and open Lichtenstein inguinal hernia repairs in India

**DOI:** 10.6026/973206300213739

**Published:** 2025-10-31

**Authors:** Shubh Narayan Ghanghoriya, Divya Sengar, Pooja Parashar, Manoj Kela, Sachin Parmar

**Affiliations:** 1 Department of General Surgery, Sri Aurobindo Institute of Medical Sciences (SAIMS), Indore,Madhya Pradesh, India; 2 Department of Community Medicine, V.K.S. Government Medical College, Neemuch, Madhya Pradesh, India

**Keywords:** Inguinal hernia, TAPP repair, Lichtenstein repair, minimally invasive surgery, postoperative outcomes

## Abstract

Inguinal hernia repair remains one of the most frequently performed surgical procedures globally, with an estimated 20 million surgeries conducted annually. Our study compares the standard method to Transabdominal Preperitoneal (TAPP) for fixing an inguinal hernia. Therefore, it is of interest to do Comparison of TAPP and open Lichtenstein inguinal hernia repairs in India. TAPP repair demonstrated significantly shorter operative time, reduced hospital stay, faster return to work and fewer postoperative complications. Both techniques were effective; however, TAPP offered superior recovery outcomes. Data support the growing preference for minimally invasive approaches in hernia surgery.

## Background:

Inguinal hernia repair remains one of the most frequently performed surgical procedures globally, with an estimated 20 million surgeries conducted annually [1, 2]. The condition demonstrates significant demographic variability, affecting approximately 1,700 per 100,000 individuals, increasing to 4,000 per 100,000 in those above 45 years of age [3]. The incidence in males is notably bimodal, peaking around age 5 and again after age 70, with a lifetime risk of 27% compared to 3% in females [4, 5]. Inguinal hernias constitute approximately 75% of all abdominal wall hernias, with indirect variants being most common across both sexes [6]. Historically, tissue-based repairs such as the Bassini and Shouldice techniques were associated with high recurrence rates, largely due to tension-induced tissue failure [7, 8]. The paradigm shifted significantly with the introduction of tension-free mesh repairs, particularly the Lichtenstein technique in 1984, which places a polypropylene mesh over the hernia defect without tension, dramatically reducing recurrence rates to below 1% [9, 10, 11-12]. This technique has since become the global standard, endorsed by leading surgical societies. Simultaneously, the advent of laparoscopic hernia repair in the early 1990s provided a minimally invasive alternative with advantages in postoperative recovery. Among the laparoscopic approaches, the Transabdominal Preperitoneal (TAPP) technique is particularly noteworthy. It facilitates access to the preperitoneal space via pneumoperitoneum, allowing mesh placement under direct vision across potential hernia sites [13, 14-15]. The TAPP method affords comprehensive anatomical visualization, potential for bilateral repair and minimized dissection compared to open surgery [16]. The procedure begins with pneumoperitoneum establishment, followed by trocar placement and peritoneal incision. Critical anatomical landmarks-including the inferior epigastric vessels, Cooper's ligament and the iliopubic tract-are visualized and the mesh is placed and covered with peritoneum to reduce adhesion risk [17, 18- 19].

Comparative studies suggest that TAPP offers advantages such as reduced postoperative pain, shorter hospital stay, faster return to work and superior cosmetic outcomes [20, 21, 22-23]. Nevertheless, TAPP requires a steeper learning curve, with up to 100 cases needed to achieve proficiency [24, 25]. It also mandates general anesthesia, limiting its use in patients with significant comorbidities, whereas Lichtenstein repair can be performed under regional or local anesthesia [26]. Tertiary care centers are uniquely positioned to compare these techniques, managing both routine and complex cases. These institutions bring together advanced surgical capabilities and structured postoperative care, thereby facilitating rigorous comparative evaluation [27, 28-29]. Despite growing literature, head-to-head comparisons between TAPP and Lichtenstein repairs yield mixed results due to heterogeneity in study design and follow-up duration [30, 31]. Meta-analyses report similar recurrence rates (0.2%-4.7%) for both techniques, with some evidence suggesting a lower risk of chronic pain with laparoscopic approaches [32, 33]. Economic considerations are critical. Although TAPP incurs higher upfront costs due to equipment and operative time, the shorter hospital stay and faster return to productivity may balance overall expenses, particularly in working-age populations [34, 35]. Quality-of-life assessments often favor TAPP in terms of pain and physical function, although the clinical magnitude of these differences remains debatable [36, 37]. Therefore, it is of interest to assess clinical outcomes associated with TAPP and Lichtenstein inguinal hernia repairs at a tertiary care center, with specific attention to operative time, postoperative complications, inflammatory response, recovery and return to work.

## Materials and Methods:

## Place of study:

The present study was conducted in the Department of General Surgery, Sri Aurobindo Medical College and Postgraduate Institute, Indore, Madhya Pradesh, India.

## Study design:

This was a prospective, randomized and comparative study.

## Study duration:

The study was conducted over a period of 17 months, from April 2021 to September 2022.

## Study population:

The study included all patients with a confirmed diagnosis of unilateral inguinal hernia visiting the institution during the study period, who were willing to participate in the study.

## Sample size:

Based on the institutional data, approximately 4 patients with unilateral inguinal hernia undergo surgical repair (either Open Lichtenstein or Transabdominal Preperitoneal (TAPP) hernia repair) each month. Consequently, a total of 34 patients were included in this study.

## Inclusion criteria:

[1] All admitted patients diagnosed with unilateral inguinal hernia that are willing to participate in the study.

[2] Patients aged between 18 and 60 years.

## Exclusion criteria:

[1] Patients unwilling to participate in the study.

[2] Patients with traumatic abdominal wall hernias.

[3] Patients diagnosed with bilateral inguinal hernia.

## Grouping:

[1] Group 1: Patients randomized to this group underwent open Lichtenstein inguinal hernia repair.

[2] Group 2: Patients randomized to this group underwent Transabdominal Preperitoneal (TAPP) inguinal hernia repair.

## Randomization:

Patients were randomized to either Group 1 or Group 2 using a lottery method.

## Methodology:

Prior to participation, all patients and/or their legally acceptable representatives were provided with a detailed explanation about the study, including the risks and benefits, the randomization process, the type of surgery being performed, potential complications and the required level of compliance. After obtaining verbal approval, written informed consent was acquired from each participant.

## Preoperative and intraoperative protocol:

Physical examination: All patients underwent a thorough physical examination.

## Investigations:

[1] Complete blood count (CBC)

[2] Prothrombin time/International Normalized Ratio (PT/INR)

[3] Serum blood urea, serum creatinine, random blood sugar, postprandial blood sugar

[4] Urine albumin, urine sugar and urine microscopy

[5] Electrocardiogram (ECG)

Preoperative anesthetic evaluation was performed for all patients. Upon confirmation that the patients were fit for surgery, they underwent the procedure as per their randomized group assignment: Group 1 patients underwent open Lichtenstein inguinal hernia repair and Group 2 patients underwent TAPP inguinal hernia repair.

## Postoperative protocol:

[1] Monitoring: Post-surgery, all vital signs were monitored. Blood investigations, including C-reactive protein (CRP) levels (as an inflammatory marker), were continuously monitored during the patients' hospital stay.

[2] Antibiotics: All patients received 5 days of intravenous antibiotics and 7 days of oral antibiotics, continued until the time of suture removal.

[3] Pain management: Pain was evaluated using the Visual Analog Scale (VAS) and wound healing, pain, complications and inflammatory response were regularly documented.

## Follow-up:

Patients were followed up on postoperative Day 1, Day 3 and Day 5. Assessments were made at each follow-up visit to monitor recovery and any emerging complications.

## Rehabilitation protocol:

Patients were advised to begin static and dynamic exercises, ambulation and heavy weight-bearing activities as tolerated.

## Data collection method:

A customized proforma was designed to collect the relevant data for the study. All information regarding the patients, including demographic details, medical history, surgical procedures and outcomes, was systematically recorded in this proforma.

## Outcome measures:

The primary outcome measures of the study were:

[1] Wound healing

[2] Postoperative complications

[3] Pain assessment (using VAS)

[4] Postoperative inflammatory response.

## Results:

In this Table 1 (see PDF), the breakdown of the patients was in two categories of 60 patients (50 percent) that went under open Lichtenstein inguinal hernia repair (Group 1) and 60 patients (50 percent) who had transabdominal preperitoneal hernia repair and both groups were of equal parts. Comparison of the demographic characteristics of patients belonging to both groups are presented in Table 2 (see PDF), which indicates that the average age was similar in the two groups, most patients falling in the age category of 41-60 years and that there was a predominant difference in the sex ratio with higher number of males in group 2 whereas no significant difference was found in the involvement of side. The comparison of surgical duration, hospital stay, and time to get back to the workplace is illustrated in Table 3 (see PDF) and shows that the Group 1 had the statistically significant longer surgical duration, longer hospital stay, and longer time to cortex with a work than Group 2, which underlines the profound difference in the time of recovery between two procedures. Table 4 (see PDF) gives an insight into post-operative pain and complications revealing that all patients reported pain on Day 0, but none reported pain on Day 1 or Day 2 and the CRP levels were significantly lower because this indicated that post-operative systemic inflammatory response was higher in Group 1, and local swelling and fever were more frequently in Group 1 and seroma in Group 2 in first 3 days. Table 5 (see PDF) contains the postoperative complications past 7 days in that the Group 1 had complications such as local swelling and fever occurring more commonly than in Group 2 and seroma formation only after 7 days in group 1 as well as no instances of urinary retention developing after 7 days in both the groups.

## Discussion:

The present study demonstrated that Group 2 (TAPP) patients experienced significantly shorter operative times (38.58 ± SD minutes vs. 60.33 ± SD minutes; P < 0.001), reduced hospital stays (3.25 ± SD days vs. 10.33 ± SD days; P < 0.001) and earlier return to work (9.60 ± SD days vs. 14.12 ± SD days; P < 0.001) compared to Group 1 (Lichtenstein). These findings align with Bhondve *et al.* [38] in terms of mean operative time and Kollampare *et al.* who reported shorter hospital stays (mean 2.0 vs. 3.0 days) and earlier return to work (50% by POD5 vs. POD10-11) in the TAPP group [39] and with Singla *et al.* who observed mean hospital stays of 2.56 days for TAPP versus 3.84 days for Lichtenstein (P = 0.001) and faster resumption of normal activities (13.8 vs. 18.6 days; P < 0.001) [40]. A systematic review and meta-analysis further confirmed an average reduction of 3.03 days in hospital stay and 5.77 days in return to work with laparoscopic approaches (P < 0.001) [41]. Regarding systemic inflammatory response, our data showed a higher proportion of patients with CRP > 0.6 mg/dL in Group 1 (15% vs. 1.7%; P < 0.05), indicating greater surgical stress in open repairs. This is consistent with Sajid *et al.* who found peak CRP values of 6.8 mg/dL in open versus 3.4 mg/dL in laparoscopic repairs [42] and with LWW reports showing significantly lower postoperative hs-CRP levels at 24 h in TEP (14.02 mg/L) compared to open repair (32.45 mg/L; P < 0.001)[26].Postoperative complications in Group 1-urinary retention, local swelling and fever-were more frequent in both early and late postoperative periods, whereas Group 2 exhibited a non-significant increase in early seroma formation. These trends mirror the findings by Sofi *et al.* [21], who reported higher rates of urinary retention (10% vs. 3.3%) and wound complications in open repairs, with lower overall early morbidity in the TAPP group and the World Journal of Plastic Surgery trial demonstrating significantly fewer wound infections, seromas and hematomas in TAPP compared to Lichtenstein (P = 0.002) [43].

## Conclusion:

TAPP hernia repair offers significant advantages over the Open Lichtenstein technique, including shorter surgical duration, reduced hospital stay, faster return to work and fewer postoperative complications. While both procedures are effective, TAPP may be the preferred choice for patients seeking quicker recovery and fewer postoperative issues. These findings contribute to the growing body of evidence supporting the use of minimally invasive techniques in hernia repair and can guide clinical decision-making in hernia surgery.
